# Enhanced Optical Absorption in Perovskite/Si Tandem Solar Cells with Nanoholes Array

**DOI:** 10.1186/s11671-020-03445-3

**Published:** 2020-11-12

**Authors:** Yawei Kuang, Yulong Ma, Debao Zhang, Qingzhu Wei, Shuchang Wang, Xifeng Yang, Xuekun Hong, Yushen Liu

**Affiliations:** 1 School of Electronics and Information Engineering, Changshu Institute of Technology, Changshu, 215500 China; 2 Suzhou Talesun Solar Technologies Co., Ltd., Changshu, 215500 China

**Keywords:** Tandem solar cell, Nanoholes array, Anti-reflection, Resonance absorption

## Abstract

Perovskite solar cells are used in silicon-based tandem solar cells due to their tunable band gap, high absorption coefficient and low preparation cost. However, the relatively large optical refractive index of bottom silicon, in comparison with that of top perovskite absorber layers, results in significant reflection losses in two-terminal devices. Therefore, light management is crucial to improve photocurrent absorption in the Si bottom cell. In this paper, nanoholes array filled with TiO_2_ is introduced into bottom cells design. By finite-difference time-domain methods, the absorption efficiency and photocurrent density in the range of 300–1100 nm has been analyzed, and the structural parameters have been also optimized. Our calculations show the photocurrent density which tends to be saturated with the increase in the height of the nanoholes. The absorption enhancement modes of photons at different wavelengths have been analyzed intuitively by the distribution of electric field. These results enable a viable and convenient route toward high efficiency design of perovskite/Si tandem solar cells.

## Introduction

Solar energy is a kind of renewable and clean energy, which is of great significance to the sustainable development of human beings. The efficiency of photoelectric conversion and the cost of preparation are the key ratios that determine the industrial application of solar cells, which directly convert light energy into electricity. At present, silicon-based solar cells are the mainstream of solar cells, accounting for 90% of the global photovoltaic market. The efficiency of silicon-based solar cells has reached 25.6%, close to the limit efficiency of Shockley–Queisser (33.7%), but the manufacturing cost remains high [1, 2]. The development of silicon-based solar cells needs to reduce manufacturing costs and improve cell efficiency.

Because of the wide energy distribution of the solar spectrum, any semiconductor material can only absorb photons whose energy value is wider than its bandgap width. Therefore, a proven approach to make better use of solar spectrum is to form a dual-junction tandem solar cell [3, 4]. In principle, Si-tandem solar cells are able to selectively absorb different parts of the solar spectrum and surpass the single-junction Shockley–Queisser. The theoretical limit efficiency of ideal two-junction silicon tandem solar cell has been reported to 46% [5–7].

Perovskite solar cells are of great photovoltaic potential, and its performance has been significantly improved in just a few years. The photoelectric conversion efficiency is 3.7% in 2009, and the efficiency has been up to 25.2% up to now [8–10]. Perovskite is also considered to be the most promising light absorbing material for the next generation of low-cost solar cells. When the bandgap width of perovskite is 1.55 eV, it can absorb photons with a wavelength less than 800 nm, while silicon with a bandgap of 1.12 eV can absorb photons with a wavelength greater than 800 nm in the solar spectrum. When the two form a tandem cell from top to bottom, their absorption spectra complement each other, which greatly improves the utilization of solar spectrum and reduces the preparation cost [11–14].

Among all kinds of perovskite/silicon tandem solar cells, the two-terminal monolithic tandem has the greatest potential because it can fabricated through directly depositing perovskite film onto a silicon bottom cell to get an integrated one. Bush et al. achieved a 23.6% efficiency on a rear emitter SHJ bottom cell with a p-i-n perovskite top cell with *E*_g_ = 1.63ev since the reduction in parasitic absorption in the front electron selective layer. Moreover, Oxford PV reached a power conversion efficiency of 28% in 2018, which further validated that the perovskite/silicon tandem has great potential to revolutionize solar cell technologies [15–17]. However, compared with silicon-based solar cells, which can reach 85% of the limit efficiency, perovskite/silicon-based tandem cells still have a lot of room for efficiency improvement. Most studies on perovskite/silicon tandem cells focus on the design of the top cell and the tunneling junction, while the bottom cell mostly adopts the textured surface or the SiN_x_ layer to improve the optical absorption [18, 19]. It is worth noting that an effective way to enhance selective absorption is to incorporate the incident light into the wave-guided mode [20]. For the above purposes, we introduce nanoholes array into the bottom subcell design. At the same time, compared with the normally textured surface, the surface of silicon nanoholes array is smoother, which is more conducive to the current matching between the top and bottom cells [21, 22].

## Methods

**Fig.1:**
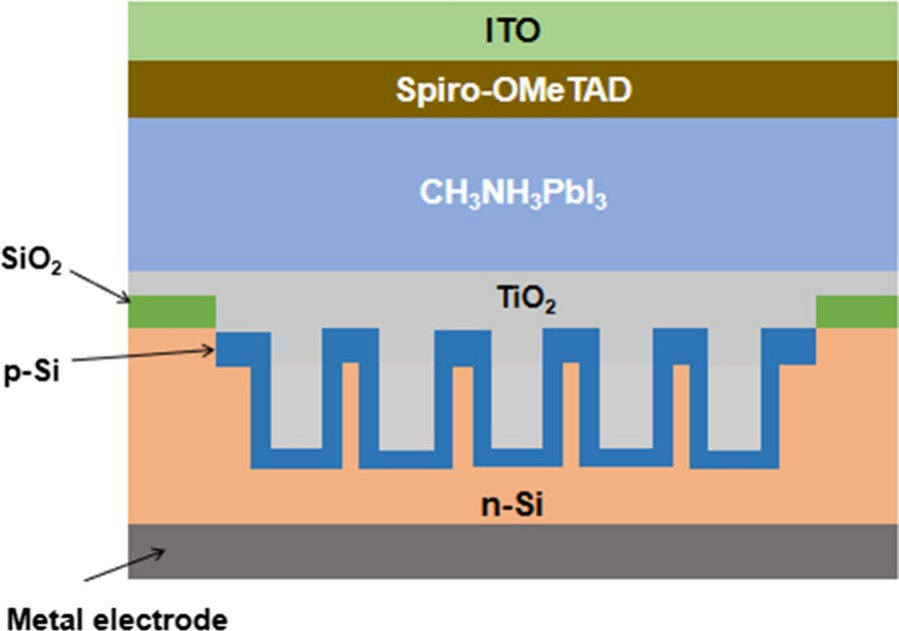
Schematic of nanoholes-structured perovskite/silicon tandems cells used in the model

**Fig. 2:**
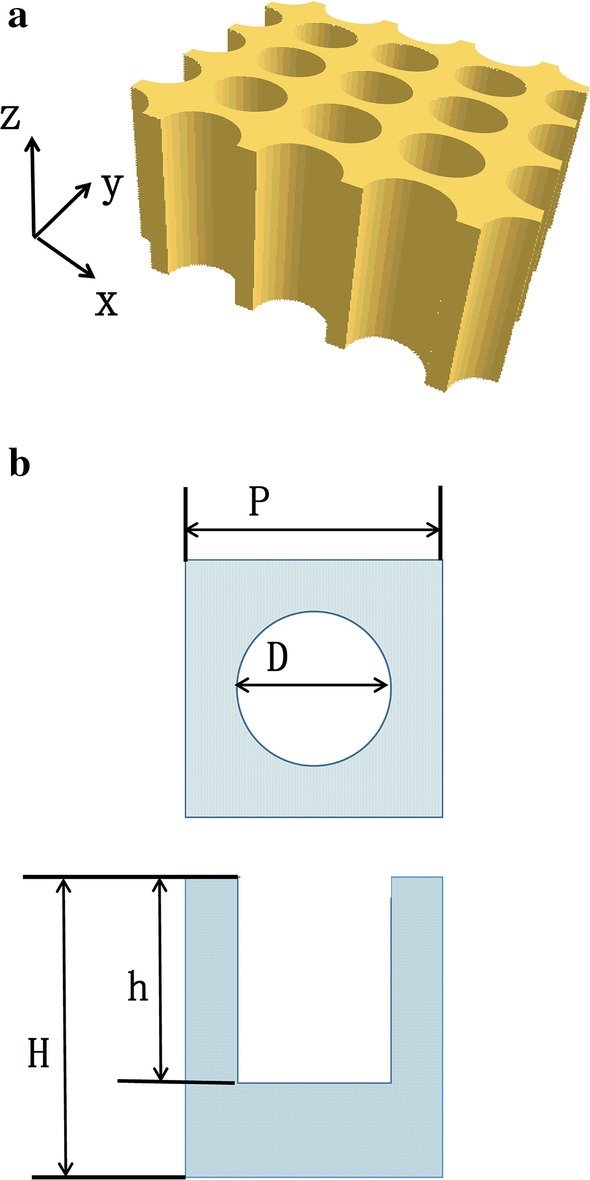
**a** Schematic of the nanoholes array used in the model. **b** 2D side view of an individual period

In this letter, we numerically study the light absorption properties of perovskite/silicon tandem device with silicon nanoholes array as bottom cells by employing the finite-difference time-domain (FDTD) method. Figures 1 and 2 show the schematic of the proposed nanoholes-structured perovskite/silicon tandems cells and the side view of an individual period, respectively.

In our model, the nanoholes array is filled with TiO_2_ as tunneling layer between two junctions. To focus the study on the optical properties of nano-structured subcell, the thickness of ITO, Spiro-OMeTAD, CH_3_NH_3_PbI_3_, SiO_2_ and TiO_2_ are fixed as 50 nm, 10 nm, 300 nm, 20 nm, 40 nm, respectively. As shown in Fig. 2, the array can be characterized by the periodicity (*P*), the diameter of nanoholes (*D*), the height of nanoholes (*h*) and total height of silicon substrate (*H*). The filling ratio is defined as $\eta = D/P$. The total height of silicon substrate *H* is fixed as 1 μm. Moreover, the optical constants of Silicon and other materials used in cell design are from F Miha’s research [23]. Periodic boundary conditions are adopted in the x and y directions and applied perfectly matched layer boundary conditions in the z direction. The light source is considered to be a planar wave source ranging from 300 to 1100 nm, perpendicular to the nanoholes array along the *z* direction.

A planar monitor above the surface of top cell is applied to register the reflectance (*R*), and a second monitor at the bottom of silicon substrate records the transmittance (*T*); the absorption (*A*) of perovskite/silicon tandems is determined by $A(\lambda ) = 1 - R(\lambda ) - T(\lambda )$. The absorption performance will be evaluated by the short-circuit current density $J_{{{\text{sc}}}}$, which is defined as [14]:

1 $$J_{{{\text{sc}}}} = \frac{e}{hc}\int_{{\lambda_{{\min}} }}^{{\lambda_{{\max}} }} {\lambda A(\lambda )\Phi (\lambda ){\text{d}}_{\lambda } }$$

where $\Phi (\lambda )$ is the solar energy density spectrum of AM1.5G, *e* is the elementary charge, *h* is Planck's constant and *c* is the light speed in vacuum. The calculation is assumed that all of the photo-generated carriers are collected by electrodes since the diffusion length of minority carriers is long enough in CH_3_NH_3_PbI_3_ and crystal silicon.

## Results and discussion

**Fig. 3:**
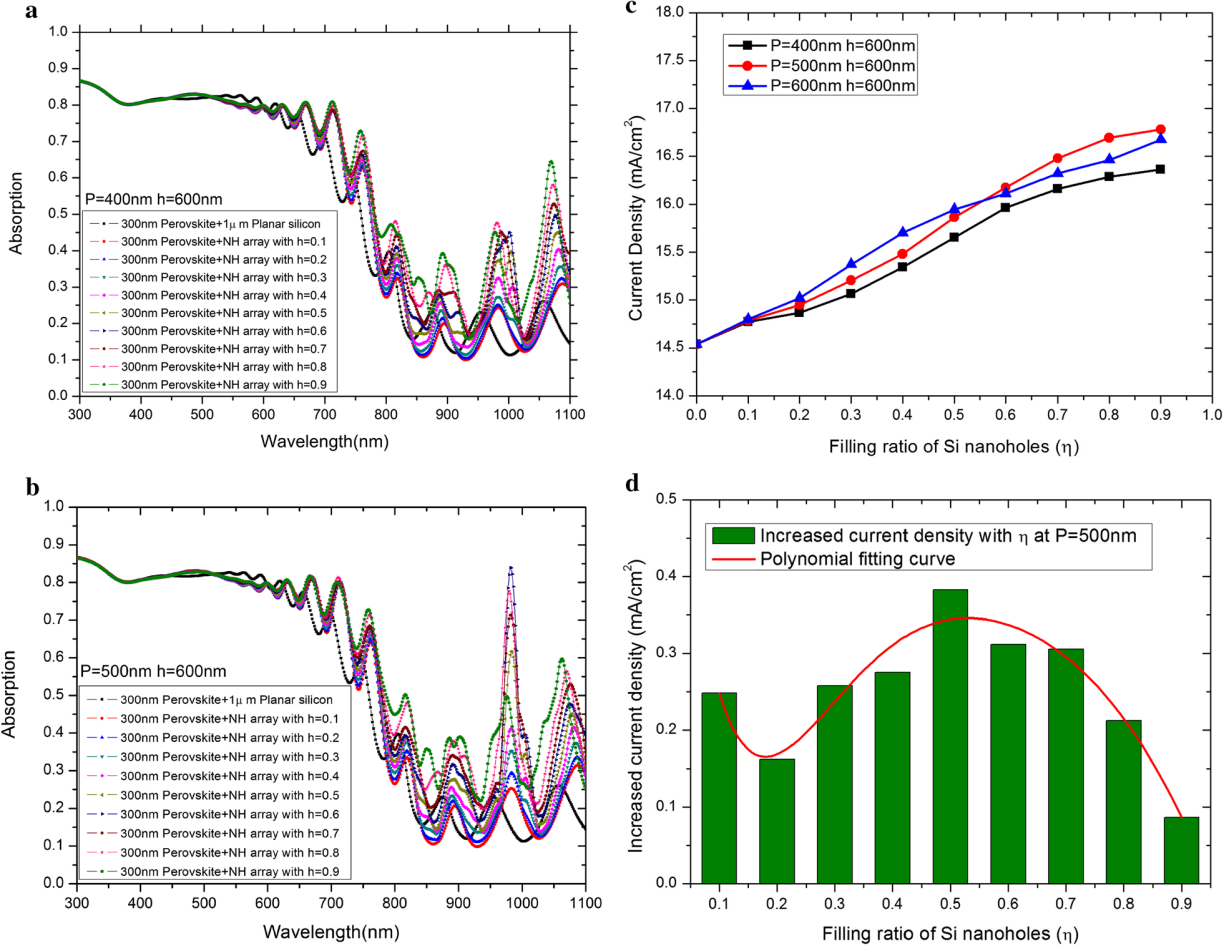
Optical characterization of tandem solar cells with nanoholes array. **a** Absorption spectra versus filling ratios at fixed *P* = 400 nm and *h* = 600 nm. **b** Absorption spectra versus filling ratios at fixed *P* = 500 nm and *h* = 600 nm. **c** Photocurrent density versus filling ratios at different periods. **d** Increasing photocurrent density versus the filling ratios under the condition of *P* = 500 nm

For the purpose of clarifying the roles that nanoholes array play on the light absorption in the tandem solar cells and in order to proper guide the design of the optical properties, we have calculated the absorption curve of nanoholes array under different filling ratios. In simulating process of experiment, a 300-nm CH_3_NH_3_PbI_3_ layer and a 1-μm silicon substrate were applied to capture photons. As are shown in Fig. 3a, b, the height of the bottom nanoholes stayed still at the figure of 600 nm against the different time periods, respectively. With the increasing value of filling ratio from 0.1 to 0.9, the absorption curve can be divided into three parts. At the beginning, the absorption presented a decreasing performance in an short wavelength range of 300–600 nm. Then, the absorption of perovskite layer was observed to form a decrease in the range of 600–850 nm, while the resonance, on the contrary, peaks at its starting point of 600 nm. The third parts fall in the range of 850–1100 nm, and it contains three absorption resonance peaks in total. Considering that the limitation of the perovskite layer’s dominance over the absorption of wavelengths could reach up to 850 nm, that value can also be considered as the threshold wavelength of CH_3_NH_3_PbI_3_ in our model.

From Fig. 3c, it can be seen that the photocurrent density and η show positively correlation, which means it will increase with the increase of η. As for the fixed period parameter, the increase in current density first appears to be in a stage with rapid growth, and the $J_{i}$ gradually enters to the saturation range where the filling ratio is greater than 0.5 due to the uneven distribution of long and short wavelengths in AM1.5G. With the increasing value of filling ratio, the absorption efficiency of silicon substrate was improved accordingly as well; however, the silicon material appears to be decreased in a single period. Therefore, the filling ratio of silicon nanoholes array should present to be an optimal value. The peak of resonance absorption reaches to the value of near 1000 nm in the spectrum, and the peak can be considered as the reaching of its maximum when the period is 500 nm compared with that of other two conditions. Figure 3d shows the curve of the photocurrent density increases along with the increasing filling ratio under the condition when *P* = 500 nm. Furthermore, the red line can be obtained through the polynomial fitting. It can be concluded that when the filling ratio reaches to exact 0.5, an inflection point will appear in the growth of the photocurrent density.

**Fig. 4:**
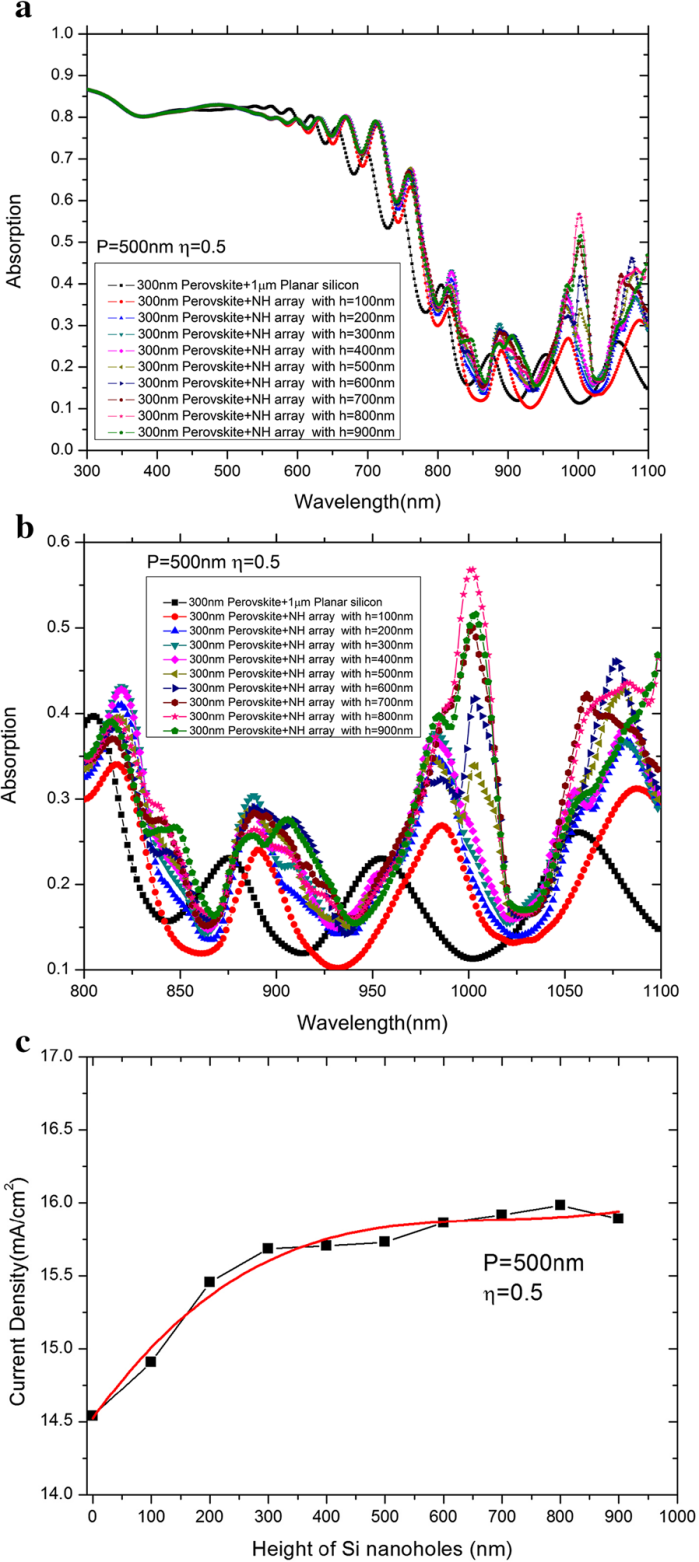
Optical characterization of tandem solar cells with nanoholes array. **a** Absorption spectra versus height at fixed *P* = 500 nm and *η* = 0.5. **b** The magnified view of the absorption spectra ranges from 800 to 1100 nm. **c** Current density versus height at fixed *P* = 500 nm and *η* = 0.5

In accordance with the above analysis, the optimized absorption parameters of the tandem cells on the basis of the nanoholes array are to be found at the period of 500 nm and the filling ratio is in the exact figure of 0.5. In order to further clarify the emission mechanism of the light absorption, the absorption spectra against the different nanoholes heights are under comparison compared in the mentioned condition. Figure 3a, b shows the changing of variation trend of spectral absorption and photocurrent density along with the increasing nanoholes heights, respectively. It can be summarized that the absorption peak at the 1000 nm wavelength shows high dependence with the height of nanoholes, while the dependence of the other two absorption peaks shown in Fig. 4b on the height of nanoholes is very weak. Such result indicates that the Mie resonance dominants the excitation. From Fig. 4c, d, the significant current density increase can be observed to climb from 14.53 to 15.68 mA/cm^2^ when the depth is less than 300 nm, and when the h values are larger than 300 nm, the value will reach an almost saturating figure. Such weak dependence on nanoholes height may be useful in terms of design as well as manufacture of nanoholes arrays in practice.

**Fig. 5:**
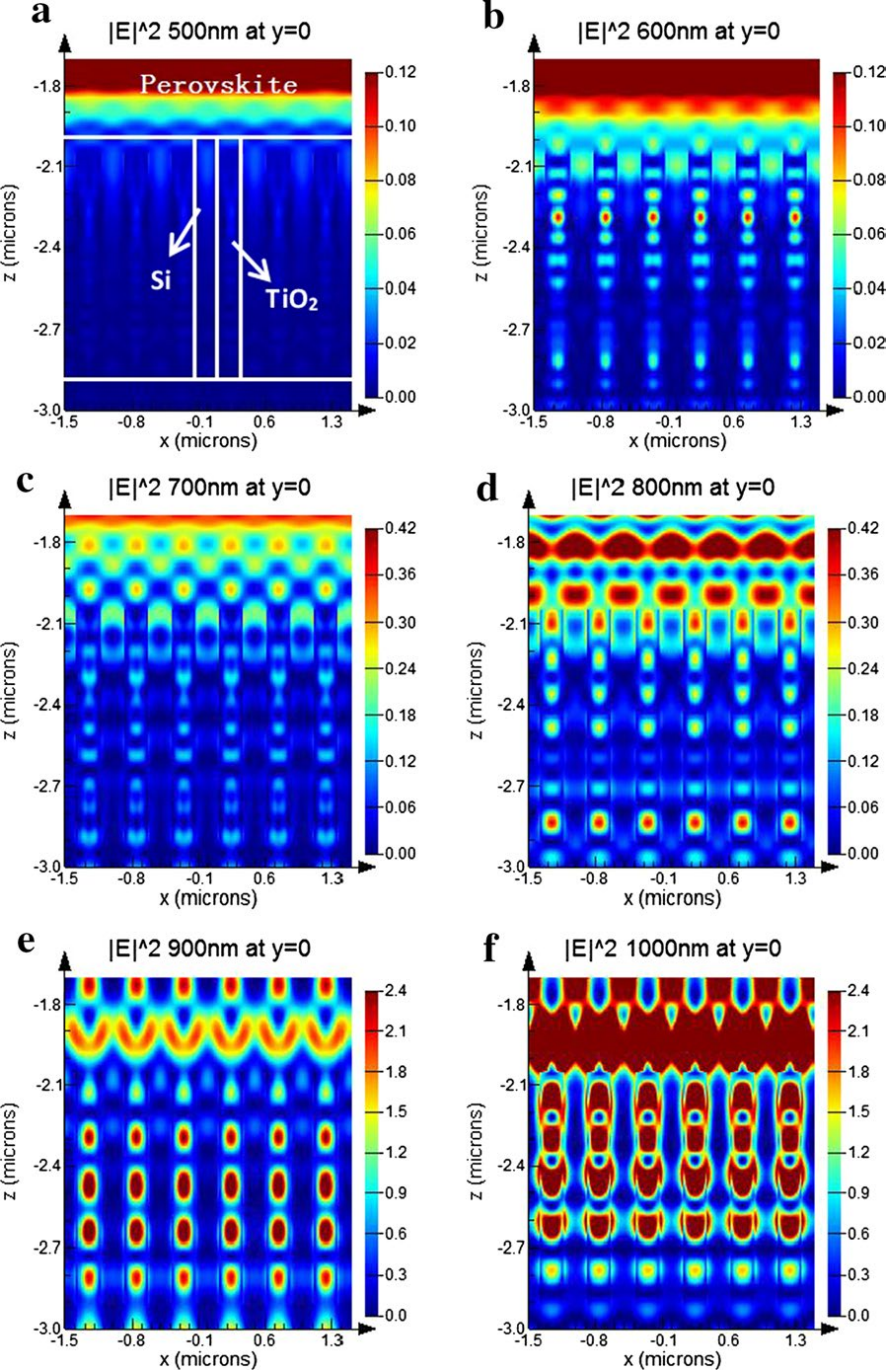
Electric field intensity distribution of tandem solar cells at fixed height 900 nm **a** wavelength at 500 nm, **b** wavelength at 600 nm, **c** wavelength at 700 nm, **d** wavelength at 800 nm, **e** wavelength at 900 nm, and **f** wavelength at 1000 nm

As a general principle, when the light wave enters into the interface structure of the tandem solar cells, the scattering and emission effects will appear. The scattering of light wave caused by the nanoholes array structure will lengthen the propagation path of photons. In order to further analyze the light absorption of perovskite/Si tandem solar cells with nanoholes array, the simulated experiments choose the cross-sectional electric field intensity distribution $(|E|^{2} )$ at 500 nm, 600 nm, 700 nm, 800 nm, 900 nm and 1000 nm wavelength, while the height is deigned to be fixed as the value of h stays at 900 nm, which is also shown in Fig. 5. The spatial profile of optical absorption per unit volume in *x*–*z* plane can be divided into three parts, which are perovskite, nanoholes array and silicon substrate. In the part of nanoholes array, the structured silicon is spaced with the TiO_2_ filled nanoholes, which is marked by dashed line in Fig. 5a.

Figure 5a, b suggests that the top cell could dominate the absorption of short wavelengths (< 600 nm); however, the anti-reflection effect produced by the nanoholes array under the wavelength of 600 nm appears to be more outstanding compered that under the wavelength of 500 nm. However, due to the lower absorption coefficient of silicon, its absorption at mid-wavelength (500–600 nm) is lower than that of the plane structure. Also thanks to the existence of the periodic nanoholes, an obvious interference effect in the top perovskite layer can also be observed, which means, the reflection of light at 700 nm and 800 nm could back into the top cells and enhance its absorption.

**Fig. 6:**
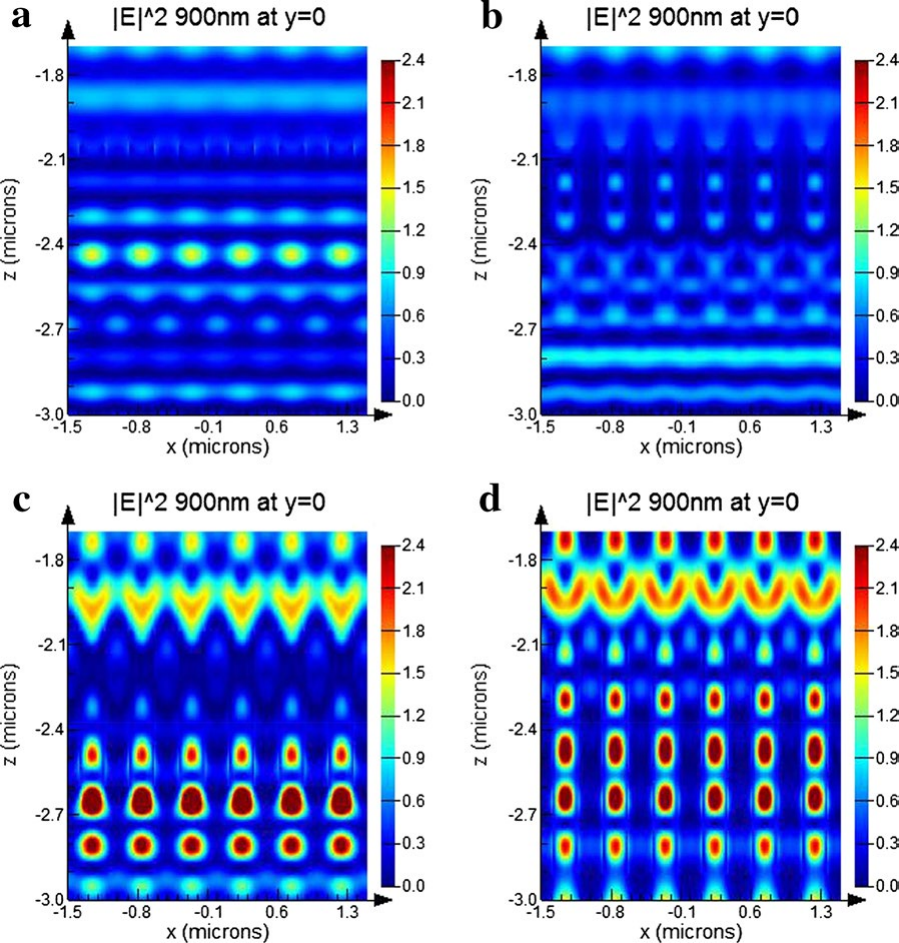
Electric field intensity distribution at 900 nm of tandem solar cells with nanoholes array versus height **a** *h* = 100 nm, **b** *h* = 300 nm, **c** *h* = 600 nm, **d** *h* = 900 nm

Provided that the perovskite has a sharp absorbing edge at 850 nm, and then, the wavelength at 900 nm and 1000 nm will be transmitted to and mainly will be absorbed by the bottom cells, as shown in Fig. 5e, f. With the filling of TiO_2_ in the silicon nanoholes array, the periodic distribution difference of refractive index leads to the bottom cells is intended to support the conducting modes which are located the electromagnetic field near the tandems, and the incident light coupling with these conducting modes leads to a prominent increase in absorption. To illustrate the feasibility of this approach, four different cases were simulated to conduct analysis under different nanoholes array height. All of these absorptions have the same nanoholes array parameters as η = 0.5 and P = 500 nm, and their incident wavelength light is fixed at 900 nm, as shown in Fig. 6. The interaction of the supported guided modes interaction is significantly enhanced along with the increasing height of the nanoholes.

## Conclusions

In summary, this article studied the combination of perovskite/silicon tandem solar cells with the nanoholes array as a practical way for a device to achieve high-efficiency tandem device. We have found that if an optimized set of nanoholes array *η* = 0.5 and *P* = 500 nm increases from the value of 14.53 mA/cm^2^ to 15.68 mA/cm^2^ when the depth of array is less than 300 nm, such device can be served as a premise for high efficiency. Then through the introduction of nanoholes array filled with TiO_2_, we have further proved that the light absorption mode of tandem cells would turn to a mixed mode with various light absorption modes. The selective reduction in short wavelength leads to the decrease in short wavelength photons absorption; however, the interference which generated the light trapping in the top cell and the index-guided light trapping in the bottom cell can functions as significantly enhance the selective absorption of the tandem. The above experimental results proved that it is a promising way to improve the absorption of perovskite/silicon tandem solar cells.

## Data Availability

The conclusions made in this manuscript are based on the data (main text and figures) presented and shown in this paper.

## Abbreviation

FDTD: Finite-difference time-domain
